# Metabarcoding for stomach‐content analyses of Pygmy devil ray (*Mobula kuhlii cf. eregoodootenkee)*: Comparing tissue and ethanol preservative‐derived DNA

**DOI:** 10.1002/ece3.4934

**Published:** 2019-02-01

**Authors:** Matteo Barbato, Toby Kovacs, Melinda A. Coleman, Matt K. Broadhurst, Mark de Bruyn

**Affiliations:** ^1^ School of Life and Environmental Sciences The University of Sydney Sydney New South Wales Australia; ^2^ National Marine Science Centre NSW Department of Primary Industries Coffs Harbour New South Wales Australia; ^3^ Fisheries Conservation Technology Unit NSW Department of Primary Industries, National Marine Science Centre Coffs Harbour New South Wales Australia

**Keywords:** DNA extraction, DNA metabarcoding, Illumina sequencing, marine predator, stomach-content

## Abstract

The application of high‐throughput sequencing to retrieve multi‐taxon DNA from different substrates such as water, soil, and stomach contents has enabled species identification without prior knowledge of taxon compositions. Here we used three minibarcodes designed to target mitochondrial COI in plankton, 16S in fish, and 16S in crustaceans, to compare ethanol‐ and tissue‐derived DNA extraction methodologies for metabarcoding. The stomach contents of pygmy devilrays (*Mobula kuhlii *cf. *eregoodootenkee*) were used to test whether ethanol‐derived DNA would provide a suitable substrate for metabarcoding. The DNA barcoding assays indicated that tissue‐derived operational taxonomic units (OTUs) were greater compared to those from extractions performed directly on the ethanol preservative. Tissue‐derived DNA extraction is therefore recommended for broader taxonomic coverage. Metabarcoding applications should consider including the following: (i) multiple barcodes, both taxon specific (e.g., 12S or 16S) and more universal (e.g., COI or 18S) to overcome bias and taxon misidentification and (ii) PCR inhibitor removal steps that will likely enhance amplification yields. However, where tissue is limited or no longer available, but the ethanol‐preservative medium is still available, metabarcoding directly from ethanol does recover the majority of common OTUs, suggesting the ethanol‐retrieval method could be applicable for dietary studies. Metabarcoding directly from preservative ethanol may also be useful where tissue samples are limited or highly valued; bulk samples are collected, such as for rapid species inventories; or mixed‐voucher sampling is conducted (e.g., for plankton, insects, and crustaceans).

## 1 INTRODUCTION

High‐throughput sequencing platforms enable generation of accurate and cost‐effective multispecies genetic assays (Mardis, 2008). Deoxyribonucleic acid (DNA) barcoding is a methodology that can provide precise and semi‐automatable species identification through the design of forward–reverse primer sets for highly conserved regions of mitochondrial (mt) DNA (Hebert et al., 2003). Combining DNA barcoding and high‐throughput sequencing has delivered a promising tool—DNA metabarcoding—to detect biodiversity in DNA extracted from materials including water (Stat et al., 2017), air (Kraaijeveld et al., 2015), soil (Drummond et al., 2015), fecal (Berry et al., 2017), and stomach‐content samples (Berry et al., 2015).

In the last decade, DNA metabarcoding of fecal matter and stomach contents has been developed, with accurate taxonomic resolution of dietary biodiversity, in attempts to infer trophic interactions among both terrestrial (Bohmann et al., 2011; Clare, Fraser, Braid, Fenton, & Hebert, 2009) and aquatic organisms (Berry et al., 2015). A fundamental step in this kind of study is DNA extraction, usually from tissue or fecal samples. However, Shokralla, Singer, and Hajibabaei (2010) proposed a novel DNA extraction methodology; successfully Sanger sequencing a universal COI barcode directly from ethanol‐derived DNA extracts. The ethanol used for sample preservation was hypothesized to be an adequate DNA carrier, thus through low temperature evaporation of ethanol and subsequent re‐suspension of the DNA pellet, the target organism's mtDNA was polymerase chain reaction (PCR) amplified and Sanger sequenced.

More recently, the same ethanol DNA extraction protocol was tested for utility in retrieving trace DNA from freshwater benthic larval communities (Hajibabaei, Spall, Shokralla, & Konynenburg, 2012) using both Sanger and 454 sequencing. Only individuals present at very low abundance (i.e., 1 individual) were not ascertained via these DNA barcoding assays. In contrast, Robertson, Minich, Bowman, and Morin (2013) compared DNA extracted from cetacean tissue of Short‐beaked common dolphin (*Delphinus delphis*), Long‐beaked common dolphin (*D. capensis*), Dall's porpoise (*Phocoenoides dalli*), Killer whale (*Orcinus orca*), Humpback whale (*Megaptera novaeangliae*), and False killer whale (*Pseudorca crassidens*). These samples were preserved for 3 to 20 years in two liquid preservatives, ethanol and 20% salt saturated dimethyl sulfoxide (DMSO). The authors were unable to detect either mtDNA or nuclear DNA from ethanol samples, while only mtDNA was amplified successfully from DMSO and Sanger sequenced.

Here, we propose that for stomach‐content analyses, DNA extracted directly from preservative ethanol could provide an accurate representation of prey diversity in a more cost‐effective and efficient manner than DNA extraction from tissue. Moreover, DNA metabarcoding may alleviate the impediment of identifying all prey DNA contained in stomach remains, if liquefied remains can be efficiently filtered in an unbiased manner. However, extracting DNA directly from stomach contents is not straightforward, because mixed‐taxon stomach samples can be several kilograms in weight, particularly in apex—(e.g., great white shark (*Carcharodon carcharias*)) and mesopredators (e.g., blacktip shark (*Carcharhinus limbatus*), Thunnus ssp.) (Pompanon et al., 2012). It can therefore be difficult to representatively subsample small amounts of (sometimes digested) tissue to recover the full diversity of ingested prey. Nonetheless, it is important to note that digestion rate plays a crucial role in the detection of tissue‐dependent extractions, sometimes causing an underrepresentation of those taxa where tissue is rapidly digested (Sousa et al., 2016). In this study, we compared the ethanol DNA extraction method (ETH) with a tissue extraction method (TIS) on the stomach‐content samples of Pygmy devilrays (*Mobula kuhlii cf. eregoodootenkee, *Müller & Henle 1841) (White et al., 2017), using three mtDNA minibarcodes: teleost (16S), crustaceans (16S), and plankton (COI). Despite recent debate over the taxonomy of *M. kuhlii cf. eregoodootenkee (*White et al., 2017)*, *the conservation status of *M. eregoodootenkee* and *M. kuhlii* are, respectively, near threatened and data deficient (IUCN 2018). Knowledge regarding habitat type is also inaccurate since the former is described as an oceanic species, and *M. kuhlii* as neritic (IUCN 2018). However, these mobulids share the same distribution across the Indo‐West Pacific Ocean. Dietary habits of both species have been scarcely described in both species, but it is generally accepted that planktonic crustaceans and possibly small fishes and cephalopods are the main prey items (Couturier et al., 2012). The rare recovery of these rays allowed us to test a novel methodology for diet research in these taxa and to fill a knowledge gap for this understudied mobulid. Since stomach‐content studies preclude a priori knowledge of the target species, we used operational taxonomic unit (OTU) analysis to test our hypothesis of no differences in recovered predator–prey relationships via metabarcoding between ethanol‐preservative and tissue‐derived DNA.

## 2 MATERIAL AND METHODS

### 2.1 Sample collection

Between January and May 2017, 31 specimens of pygmy devil rays were identified and collected by the New South Wales Department of Primary Industries (NSW DPI) from five bather protection nets deployed off northern New South Wales, Australia. After recovery, the individual specimens were transported by vehicle at ambient temperature to NSW DPI Center in Ballina (NSW, Australia) and immediately frozen at −20°C to preserve the specimens, prior to bulk assessment of all animals, where the animals were collectively defrosted and necropsied following Broadhurst, Laglbauer, Burgess, and Coleman (2018). From visual inspection of stomach contents, it was possible to distinguish small fragments of prey remains, some of which were identified as sandy sprat (*Hyperlophus vittatus*). The stomach contents of 27 of the 31 specimens, which were relatively undegraded, were preserved in 50 ml of 100% ethanol to avoid further degradation of the samples.

### 2.2 DNA extraction

In September 2017, 1.5 ml aliquots of ethanol were taken from each of 10 pygmy devil ray stomach‐content samples without disturbing the settled tissue matter. For each sample, five technical replicates were taken to increase the final amount of DNA. These samples, along with five negative controls, were heated at 56°C until the ethanol had completely evaporated (Shokralla et al., 2010). Microbiology grade DNAse‐free water was added, and the samples were left at ambient temperature overnight. The samples were then vortexed to encourage resuspension, and technical replicates were combined. Tissue was also taken from the same 10 samples for comparison. We attempted to subsample the tissue as representatively as possible, particularly subsampling larger tissue pieces in an attempt to avoid swamping the sample with a single taxon. The DNA was extracted using a Qiagen DNeasy blood and tissue kit (Qiagen, Germany) following the manufacturer's instructions (Qiagen, 2006), with 10 min incubation after addition of elution buffer, instead of 1 min, to ensure adequate elution. The DNA extraction was carried out in a pre‐PCR laboratory to minimize the risk of contamination. Filter tips were used, and negative controls were included in all stages of the laboratory workflow. Clean room protocols were followed, with extensive bleaching of the work areas and UV‐treatment of equipment, wherever possible.

### 2.3 Positive controls

Positive controls are a vital inclusion in any metabarcoding PCR because they allow distinction between problems regarding PCR conditions and sample DNA extractions, and can also be used to troubleshoot and calibrate downstream metabarcoding issues (Deiner et al., 2017). Here, positive controls were sampled for DNA on a subsequent day to the stomach‐content samples to avoid cross‐contamination and comprised the following: white banana prawn (*Penaeus merguiensis, *de Man 1888), brown tiger prawn (*Penaeus esculentus, *Haswell 1879), deepwater flathead (*Platycephalus conatus, *Waite & McCulloch 1915), and eastern school whiting (*Sillago flindersi, *McKay 1985). These specimens were chosen because they were likely to amplify using the primers used in this study and were accessible from a local fish market. Sections of tissue were removed and the same DNeasy tissue extraction kit was used. Positive controls were tested using routine universal primers HCO2198 and LCO1490 for prawns (Folmer, Black, Hoeh, Lutz, & Vrijenhoek, 1994), and Fish F1 and Fish R2 for fish (Ward, Zemlak, Innes, Last, & Hebert, 2005) to confirm the extraction protocols and PCR assays were working.

### 2.4 Metabarcoding assay

Three previously designed and tested group‐specific barcode primers were selected for teleosts, crustaceans, and plankton, targeting 16S mtDNA (teleost and crustacean) and the cytochrome c oxidase subunit I gene (plankton) (Table 1). The DNA extract concentrations were measured using a NanoDrop. The ratio of 260 and 280 nm absorbance was recorded to examine protein inhibition in the DNA extracts (Zarzoso‐Lacoste et al., 2013). The PCR was performed following the protocol recommended by Taberlet, Bonin, Zinger, and Coissac (2018) using 10 µl of AmpliTaq Gold 360 DNA Master Mix (Thermo Fisher Scientific, USA), 2 µl of forward and reverse primer, 0.16 µl bovine serum albumin to decrease the risk of PCR inhibition, 2 µl of genomic DNA, and 4 µl of DNA‐free water. After activation at 96°C for 10 min, each PCR run consisted of 35 cycles: 30 s at 96°C denaturation, 30 s at 50°C hybridization, and 1 min at 72°C elongation (Taberlet et al., 2018). The PCR products were tested on a 2% Agarose gel to confirm the successful amplification of the target amplicon of correct size, while subsequent PCR assays were undertaken using each primer with the addition of the appropriate Illumina overhang adaptors (forward overhang 5’TCGTCGGCAGCGTCAGATGTGTATAAGAGACAG [sequence‐specific primer] 3’ and reverse overhang 5’GTCTCGTGGGCTCGGAGATGTGTATAAGAGACAG‐ [sequence‐specific primer] 3’) using the same PCR conditions as above. Two PCR replicates for each primer set were conducted for each sample. The PCR products were sent to the Ramaciotti Centre for Genomics at The University of New South Wales (Sydney, Australia) for PCR cleanup using AMPure XP beads and Index PCR. This process involves attaching Illumina unique MIDs (Multiplex IDentifiers) to the amplicons of each sample, using primers that bind to the Illumina overhang adaptors. After two repetitions of PCR clean‐up, the libraries were quantified, normalized, and pooled before paired‐end sequencing was performed on an Illumina MiSeq platform. Because single step (indexing) PCR has been shown to significantly bias sequence abundance, we chose to use the Illumina recommended two‐step procedure for metabarcoding (O'Donnell, Kelly, Lowell, & Port, 2016).

**Table 1:** Minibarcode assay, Primer sequence, and PCR conditions

Barcode	Target taxa	Primer	Sequence	Amplicon length (bp)	*T*_m_ (°C)	Reference
16S	Fish	Fish 16S Forward/d	5′ GACCCTATGGAGCTTTAGAC 3′	~200	54	Berry et al. (2017)
		16S reverse/d	5′ CGCTGTTATCCCTADRGTAACT 3′			Deagle et al. (2007)
COI	Plankton	Minibar‐Mod‐F	5′ TCCACTAATCACAAAGAYATYGGYAC 3′	~127	52	Berry et al. (2015)
		Minibar‐Mod‐R	5′ AGAAAATCATAATRAANGCRTGNGC 3′			
16S	Crustacean	Crust16S_F(short)	5′ GGGACGATAAGACCCTATA 3′	~170	51	Berry et al. (2017)
		Crust16S_R(short)	5′ ATTACGCTGTTATCCCTAAAG 3′			

### 2.5 Bioinformatic pipeline

All Illumina reads went through quality filtering comprising two main steps. The first involved pairing, merging, and trimming off the forward–reverse primers using Geneious software (Kearse et al. 2012). Reads were discarded if primers were not present, not exactly matching the primer nucleotide lengths, or if reads exceeded the mismatch number of ambiguities according to the primer sequences. The second quality‐filtering step was executed through USEARCH. Reads were removed if containing ≥1 ambiguities, maximum error above 0.5, were less than 50 bp in size or exceeding the expected amplicon length (Table 1). Sequences were dereplicated into groups of unique sequences. Singleton groups were discarded (Edgar, 2010).

Following USEARCH OTU pipeline recommendations, we conducted an OTU analysis that consisted of clustering sequences with 97% similarity through the execution of the UPARSE algorithm (Edgar 2010). Moreover, this process removed any sequencing errors, PCR artefacts, chimeras, and low‐abundance clusters <0.75% of the total number of unique sequences identified within the sample. The OTUs from ETH and TIS extractions were inspected to identify common OTUs using the USEARCH *search_exact* and *search_global *function, respectively, with 100 or 80% identity thresholds. All ETH and TIS reads were separately mapped using the USEARCH command *otutab* that allowed visualizing the number of reads for each OTU within each sample.

Subsequently, BLASTn (Basic Local Alignment Search Tool, National Center for Biotechnology Information's (NCBI) GenBank nucleotide database, Altschul, Gish, Miller, Myers, & Lipman, 1990) was performed for searching OTUs with default parameters and a reward of 1 through the use of the high‐performance computer facility Artemis at The University of Sydney. Outputs were imported into MEGAN6 (MEtaGenome ANalyzer; Huson et al., 2016) to inspect taxonomic identification using the LCA parameter set as a minimum bit score of 150.0 and the top 5% matches. Only fully matching queries were considered in the assignment of taxa. Additional information on identified taxa was obtained from the Atlas of Living Australia (ATLAS) (2018). In all cases, when OTUs were examined further for taxonomy, they made biological (potential pygmy devilray prey items) and geographical (Australian east coast marine distribution) sense. In the case of the 16S fish assay, the main prey item identified by metabarcoding was also the dominant prey item in visual inspections of gut contents (sandy sprat, *Hyperlophus vittatus*). Particular attention was paid to exogenous contaminations, absent in the controls but present in at least one sample. Specifically, if the distribution of a suspicious taxon, examined by ATLAS, was not present in the area of study or it was impossible to be pygmy devilray prey that OTU was discarded.

### 2.6 Statistical analysis

R Studio was utilized for all statistical analysis (RStudio Team, 2015). Data normality was evaluated by Shapiro (Shapiro and Wilk 1965) and Levene (Levene, 1960) tests on DNA concentration and absorbance ratio data. Both were normally distributed (Shapiro test *p*‐value <0.05 and Levene test *p* value >0.05) so a paired *t*‐test was executed. The normality of common OTU reads was evaluated in the same manner. As these values were not normally distributed (Shapiro test *p* value >0.05), the non‐parametric Wilcoxon test (Wilcoxon, 1945) was executed in order to inspect the similarity of the read distribution of common OTUs between the TIS and ETH methods for the three minibarcodes (16S fish, 16S crustacean and COI plankton), using R Studio.

## 3 RESULTS

### 3.1 DNA extraction

**Figure 1:**
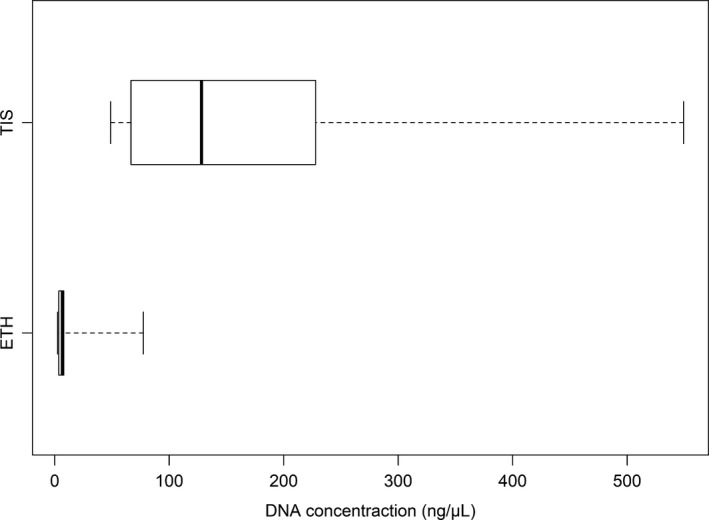
DNA concentration (NANODROP) after extraction (*t*‐value = 3.47; *p*‐value = 0.007). Bars show the range of DNA concentration

**Figure 2:**
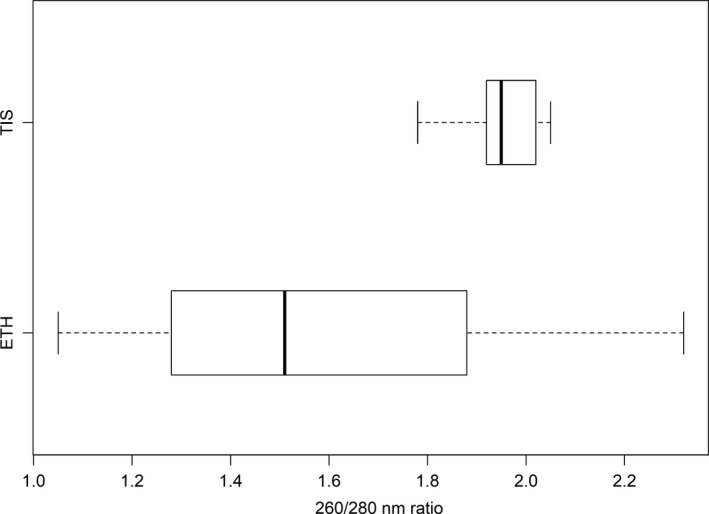
Range of inhibition according to 260/280 NM ratio (*t*‐value = 2.56; *p*‐value = 0.03). Here values of 1.8 to 2.0 indicate little inhibition

The initial DNA concentrations derived from TIS were significantly greater than those for the ETH (*t*‐value = 3.47; *p*‐value = 0.007; Figure 1). Based on the 260/280 absorbance ratio (Figure 2), TIS DNA measurements ranged between 1.78 and 2.00, showing low protein inhibition whereas ETH‐retrieved DNA had significantly greater inhibition levels (1.05–2.50) (*t*‐value = 2.56; *p*‐value = 0.03).

### 3.2 Metabarcoding results and Operational Taxonomic Unit analysis

Executing BLASTn helped to identify exogenous contaminations and non‐target taxa such as Amniota and Bacteria, which were confidently excluded from further analyses. The negative controls had low levels of endogenous contamination, and those OTUs were removed from both sample sets. The positive controls accurately identified the target species (white and brown banana prawns, deepwater flathead, and eastern school whiting), as well as other market fish (tuna, salmon, and conger eel for fish 16S), illustrating the universal fish primer efficiency and the sensitivity of the metabarcoding approach. The positive controls were purchased from a fish market with multiple species alongside one another, reflecting the sensitivity of the metabarcoding approach in detecting these likely contaminants.

**Figure 3:**
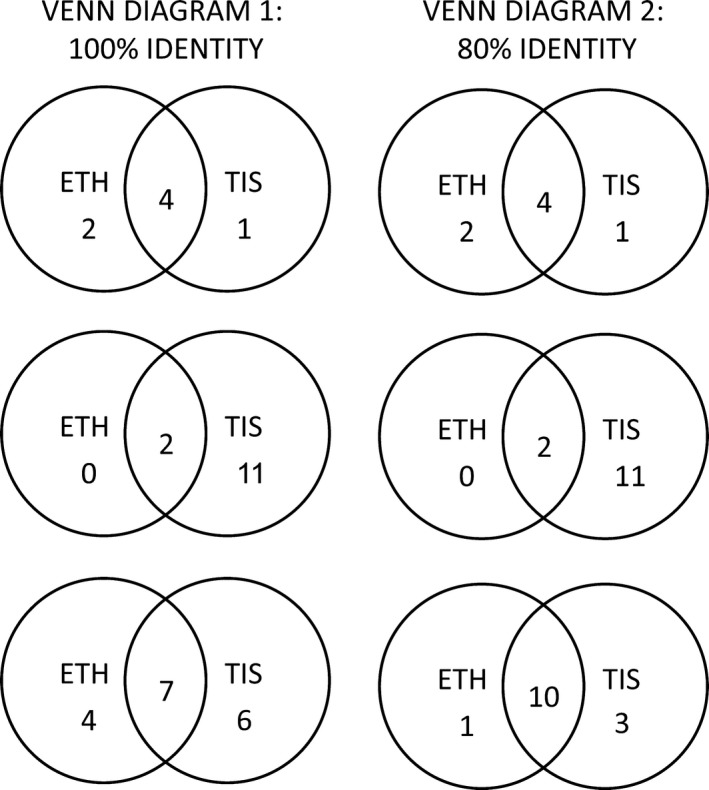
OTUs matched using USEARCH function search_exact and search_global. From top to bottom, 16S Fish, 16S crustacean and COI Plankton. Ethanol preservative‐derived OTUs (ETH) and tissue‐derived OTUs (TIS)

The OTU analysis detected 11 and 13 OTUs in plankton COI, 6 and 5 OTUs in the fish 16S assay, and 2 and 13 OTUs in the crustacean 16S assay, for ETH and TIS, respectively. The distribution of reads for common OTUs across samples was similar between ETH and TIS (Table 3). The TIS method retained the greatest number of OTUs in plankton COI and crustacean 16S (Figure 3). In contrast, for the fish 16S assay, ETH showed one more OTU than TIS. In addition, we tested an 80% identity threshold to explore whether a lower similarity could give the same number of common OTUs. Our reasoning was that “false” OTUs could be generated when sequence lengths differed, or when high levels of intraspecific genetic diversity were present, or through sequencing error or other such biases. Although the 80% cut‐off contributed to a larger number of common OTUs than for the 97% cut‐off, it still indicated that TIS recovered more OTUs than the ETH (Figure 3).

## 4 DISCUSSION

While DNA metabarcoding is not exempt from technical uncertainties such as PCR‐bias and sequencing errors—which should not be underestimated (Bohmann et al., 2011; Berry et al. 2017; Albaina et al., 2016)—this DNA metabarcoding methodology can provide an improved analysis of feeding habits compared to visual inspection, in line with other studies (Table 2). However, this methodology alleviates but does not resolve the underrepresentation issue regarding prey tissues that have different digestion rates. To the best of our knowledge, however, no studies have tested ethanol, used to preserve stomach‐content samples, as a suitable DNA carrier in the application of metabarcoding for dietary analysis. Our results show that it is possible to extract, amplify, and detect DNA by metabarcoding directly from ethanol in line with previous genetic applications such as biomonitoring surveys (Hajibabaei, Shokralla, Zhou, Singer, & Baird, 2011; Hajibabaei et al., 2012) and DNA presence–absence using Sanger sequencing (Shokralla et al., 2010). Nevertheless, when comparing OTU richness between the two methods, ethanol does not appear to recover the full range of prey. Although it was possible to detect common OTUs in both the ETH and TIS, and overall the read distributions of common OTUs were statistically equivalent for both methods (*p* value >0.05, Table 3), the TIS recovers more unique OTUs for plankton COI and crustacean 16S. Thus, ethanol‐derived DNA may not inform on the entire spectrum of feeding habits of a species and could potentially be misleading when rare or low biomass prey are consumed.

**Table 2:** Methodology summary of metabarcoding studies on marine organism diets using stomach‐content samples

Study	Organism	DNA extraction kit	Inhibition removal	qPCR	Sequencer
Albaina et al. (2016)	European sardine (*Sardina pilchardus*) and European sprat (*Sprattus sprattus*)	Salt extraction protocol	Addition of bovine serum albumin	As proxy of prey abundance and sensitivity of 18S v9 assay	Pair‐end Illumina MiSeq
Berry et al. (2015)	Common jack mackerel (*Trachurus declivis*), gemfish (Rexea solandri), tiger flathead (Platycephalus richardsoni), reef ocean perch (Helicolenus percoides), jackass morwong (Nemadactylus macropterus), Pink ling (Genypterus blacodes), blue warehou (Seriolella brama) and john dory (Zeus faber)	Qiagen DNeasy blood and tissue kit primer	No	Yes, to measure concentrations of starting DNA template	454 GS Junior (Roche)
Fernández‐Álvarez, Machordom, García‐Jiménez, Salinas‐Zavala, & Villanueva (2018)	Flying squids (family Ommastrephidae)	QIAamp DNA Investigator Kit	Included in the extraction kit	no	Pair‐end MiSeq Illumina
Harms‐Tuohy et al. (2016)	Lionfish (*Pterois volitans*)	Qiagen DNeasy Blood and Tissue kit	No	No	llumina MiSeq platform
Leray et al. (2013)	Cardinal fish* (*Nectamia savayensis*),* soldierfish (*Myripristis berndti*) and squirrelfish (*Sargocentron microstoma)*	Qiagen DNeasy Blood and Tissue kit	MOBIO PowerClean DNA cleanup kit	No	Roche 454 FLX sequencing
JakubavičiūtėBergström, Eklöf, Haenel, & Bourlat (2017)	Three‐spined stickleback (*Gasterosteus aculeatus*)	UltraClean^® ^Tissue and Cells DNA Isolation Kit(MOBIO Laboratories)	Included in extraction kit	No	Pair‐end Illumina Mi seq
Siegenthaler, Wangensteen, Benvenuto, Campos, & Mariani (2018)	Brown shrimp *(Crangon crangon)*	PowerSoil® DNA Isolation Kit (MoBio laboratories)	Included in extraction kit	No	Pair‐end Illumina 2x250
Sousa et al. (2016)	Sun fish (*Mola Mola*)	JetQuick DNA Kit (GENOMED)	No	No	Roche 454 GS FLX
Yoon et al. (2017)	Antarctic toothfish (*Dissostichus mawsoni*)	AccuPrep^®^ genomic DNA extraction kit	Included in extraction kit	No	Pair‐end Illumina Miseq

**Table 3:** The distribution of metabarcoding reads of the common operational taxonomic units (OTUS) between ETH and TIS for the three minibarcodes (16S Fish, 16S crus, and COI Plankton) was evaluated by using wilcoxon test (Wilcoxon 1945). When the *p*‐value is lower than 0.05 (shown in BOLD), the two methods show different read distributions

Sample	16S Fish		16S Crus		COI Plan	
	*V* value	*p* value	*V* value	*p* value	*V* value	*p* value
1	3	0.37	0	1	3	1
2	0	0.18	0	0.5	4	**0.04**
3	6.5	0.71	0	1	0	0.1
4	0	0.37	0	0.5	5	1
5	1	1	0	1	1.5	1
6	3	0.37	0	0.5	5	1
7	1	1	1	1	0	0.37
8	3	1	0	0.5	6	0.4
9	0	0.37	0	0.5	0	0.37
10	2	0.37	0	1	11	1

In this study, we used a standard DNA extraction kit for the TIS samples, foregoing any attempts to remove inhibition (besides using BSA in the PCR assays) from the samples such as qPCR and serial dilutions, to preclude biasing the downstream analyses. Even so, the TIS extractions were less inhibited than the ETH indicated by the 260/280 absorbance ratio that reflects protein inhibition of samples (Figure 2). In a study of DNA extraction performance in stomach samples, Zarzoso‐Lacoste et al., 2013) confirmed a better amplification yield with low DNA concentrations (8.2–71 ng/ml) and low protein inhibition (the 260/280 ratio being between 1.80 and 2.00), than high DNA concentrations (70.9–408 ng/ml) with absorbance ratios below 1.80, measured by Nanodrop.

The performance of the three minibarcodes was generally successful, and a multiple barcode approach can improve the outcome of metabarcoding studies (Berry et al., 2017). However, COI has been debated to not be particularly suited for short amplicon‐based applications because it does not contain sufficiently conserved regions, despite the availability of a species‐specific reference database (Deagle, Jarman, Coissac, Pompanon, & Taberlet, 2014). To overcome the need for a universal barcode, some researchers have replaced COI with an 18S nuclear DNA barcode in diet analysis (Albaina et al., 2016) and environmental DNA surveys (Stat et al., 2017). In fact, considering that bacterial sequences (also found in the raw data analysis and removed from OTU analysis), unidentified “no hits” and other eukaryotic taxa were detected by the COI barcode, this reflects its universal detection ability (Somervuo et al., 2017). For our results, the high overall number of COI OTUs could be due to COI barcode limitations, that is a large number of unidentified “no hits”.

Clearly, the period of digestion, and inhibition due to bacteria, digestive enzymes etc., are two crucial variables in dietary assays (Zarzoso‐Lacoste, Corse, & Vidal, 2013). The longer prey DNA goes through digestive phases, the more inhibited it is likely to be, effectively influencing its quantification and amplification (Taberlet et al., 2018). Nonetheless, gut‐derived prey DNA is thought to be of better quality than scat‐derived prey DNA since digestion is at its initial phase. As one example, Kamenova, Mayer, Coissac, Plantegenest, and Traugott (2017) noted gut DNA was detectable for longer than scat DNA after periods of feeding and starvation in the predatory carabid beetle (*Pterostichus melanarius)*. In fish stomachs, DNA persistence has been recorded ranging between 16 and 24 hr post digestion using mid‐throughput sequencing (Carreon‐Martinez et al., 2011). However, because the stomach likely contains bacteria and co‐extracted substances, DNA can still certainly be affected by inhibition impairing PCR amplification (Zarzoso‐Lacoste et al., 2013). Various commercial DNA extraction kits enable the reduction or removal of inhibition. Most published studies describing marine organism diets using stomach‐content samples either included an analysis step, or extraction kit that reduced inhibition (Table 2). An example of dealing with PCR inhibitors in dietary studies involves scat samples, which are less invasive and far easier to collect than stomach samples (Berry et al., 2017; Hardy et al., 2017). Scat DNA was often found to be highly inhibited and degraded. Quantitative PCR (qPCR) is a key step that facilitates determining an appropriate dilution to reduce inhibition levels for downstream analysis, and for estimating whether sufficient target DNA is obtained from the DNA extraction. In fact, coupling qPCR and high‐throughput sequencing is thought to be the best option for in‐depth diet analysis when using scat samples (Murray et al., 2011). However, studies on stomach samples of marine organisms did not always include qPCR before NGS sequencing (Table 2). Although we did not use the qPCR approach here, it is clear that qPCR provides clear benefits for metabarcoding studies.

The study organisms’ (Pygmy devil ray) diet has not been well studied. Nevertheless, dietary research on mobulids has described the group as mostly planktivorous, feeding on various zooplankton and small fish (Couturier et al., 2012). Although the techniques in use, that is stable isotope and fatty acid profiling, and visual identification are generally accepted, most existing methods have failed to resolve higher taxonomy in prey identification (Pompanon et al., 2012; Berry et al., 2015). Since the collection of scat samples from many marine organisms are particularly challenging, dietary studies rely on stomach samples, which in turn are dependent on fishery and fishery‐independent sampling or strandings. Moreover, the ethanol‐retrieved DNA methodology could be applied where stomach contents are in a liquefied state or tissue is not available. It is clear that the DNA metabarcoding approach has already provided new insights into the dietary habits of several marine organisms (Table 2). This approach has been implemented in generalist feeders resulting in improved resolution compared to visual inspection. For instance, a dietary study of the Lionfish (*Pterois volitans*) contributed to identifying 39 species from digested liquiform samples, whose identification would be difficult using traditional methods (Harms‐Tuohy, Schizas, & Appeldoorn, 2016). Interestingly, stomach‐content analysis of the brown shrimp (*Crangon crangon),* a generalist/scavenger feeder, has been used recently as a natural sampler to monitor estuarine biodiversity through DNA metabarcoding (Siegenthaler, Wangensteen, Soto et al., 2018). Therefore, future dietary studies should consider the importance of fine‐tuning a methodology to allow accurate recovery and identification of the full range of prey.

## 5 CONCLUSIONS

Studying trophic dynamics via high‐throughput sequencing requires an efficient low‐cost methodology that accurately recovers, identifies, and characterizes all ingested prey—in terms of both taxonomic diversity and ideally, levels of biomass (Taberlet et al., 2018). The methodology implemented for dietary studies of marine organisms varies according to the study taxon and the sample condition (Table 2). Even though this study investigated only one animal species, we suggest that DNA extractions from stomach‐content tissue are more reliable than extractions directly from preservative ethanol but comparing these two methods in other species is required in order to verify whether this finding is universal. Ethanol‐based methods can still facilitate a rapid overview of diet, and such methods may be applicable for liquefied stomach contents, using ethanol for resuspension when tissue samples are not available. The method might also be applied to bulk mixed taxonomic vouchers (e.g., bulk insect/plankton/crustacean collections preserved in ethanol)—a method routinely used in rapid biodiversity inventories.

Future research warrants testing ethanol extraction coupled with inhibition removal kits to determine if ethanol performs equally well as tissue. However, this may entail an increase of material costs and laboratory work. Moreover, as qPCR has an important role in ensuring robustness of the methodology, its effectiveness should be evaluated when DNA extraction kits include an inhibition removal step. The application of multiple DNA metabarcoding assays is certainly a promising approach for taxonomic studies on the feeding habits of marine organisms. However, this type of study will always be constrained by spatial and temporal coverage. Integrating the metabarcoding approach with isotope analysis and fatty acid profiling would improve resolution for long‐term dietary studies.

We conclude that when stomach‐content samples are available, researchers should consider which methodology is appropriate according to the sample status (e.g., level of degradation) and current knowledge of the host diet (e.g., generalist or specialist feeder). However, assessing diet only via the visual identification of ingested prey may underestimate taxonomic diversity. It is essential to integrate new high‐throughput approaches into dietary studies to inform fishery management and marine conservation; implicit among which is identifying appropriate, low‐cost metabarcoding methodologies to accurately assess taxonomic composition.

## CONFLICT OF INTEREST

None declared.

## AUTHOR CONTRIBUTIONS

M.d.B., M.A.C., and M.K.B. conceived the study. M.K.B. and M.A.C. collected the samples. M.B., T.K., and M.d.B. designed and conducted the experiments. M.B. analyzed the data. M.B. and M.d.B. wrote the paper. All authors contributed intellectually to the interpretation of the results and writing of the manuscript.

## Data Availability

https://doi.org/10.5061/dryad.288vb66
